# Epidural adipose tissue-derived mesenchymal stem cell activation induced by lung cancer cells promotes malignancy and EMT of lung cancer

**DOI:** 10.1186/s13287-019-1280-3

**Published:** 2019-06-13

**Authors:** Yan Wang, Yijing Chu, Xianfeng Ren, Hongfei Xiang, Yongming Xi, Xuexiao Ma, Kai Zhu, Zhu Guo, Chuanli Zhou, Guoqing Zhang, Bohua Chen

**Affiliations:** 1 Department of Orthopaedic Surgery, The Affiliated Hospital of Qingdao University, 59 Haier Road, Qingdao, 266061 China; 2 Department of Obstetrics and Gynaecology, The Affiliated Hospital of Qingdao University, Qingdao, China

**Keywords:** Metastatic epidural spinal cord compression, Adipose-derived mesenchymal stem cells, Pre-metastatic niches, Lung cancer, STAT3 signalling pathway

## Abstract

**Background:**

Spinal metastasis is a major challenge in patients with advanced lung cancer, but the mechanisms in the organotropism of metastasis are still unclear. Adipose-derived mesenchymal stem cells (ADSCs) exhibit cancer-promoting properties that influence the tumour microenvironment; however, there is no research on ADSCs from epidural fat thus far.

**Methods:**

In this study, we isolated and identified ADSCs from epidural adipose tissue for the first time. We examined the activation of epidural ADSCs treated with lung cancer cell-conditioned medium by immunohistochemistry, western blot and qRT-PCR assays. The expression of interleukin (IL)-6 family cytokines in the supernatants of ADSCs were evaluated by enzyme-linked immunosorbent assay. The effects of epidural ADSCs on the growth and invasion of lung cancer cells were evaluated with the CCK-8 and Transwell assays. The expression of signal transducer and activator of transcription 3 (STAT3), matrix metalloprotease and epithelial-mesenchymal transition markers were measured by western blot assays.

**Results:**

Our results showed that ADSCs treated with lung cancer cell-conditioned medium expressed higher levels of the myofibroblast marker α-smooth muscle actin and fibroblast activation protein than ADSCs cultured alone. Then, we found that lung cancer cells induced ADSCs to secrete high levels of IL-6 family cytokines and activate the STAT3 signalling pathway. Moreover, activated epidural ADSCs exhibited the ability to promote lung cancer cell proliferation and invasion by elevating matrix metalloprotease expression and epithelial-mesenchymal transition in cancer cells. Furthermore, blocking IL-6 can counteract the differentiation and tumour-promoting effects of ADSCs.

**Conclusion:**

Our results suggest that ADSCs respond to lung cancer cells and are involved in the crosstalk between primary tumours and pre-metastatic niches in epidural fat.

**Electronic supplementary material:**

The online version of this article (10.1186/s13287-019-1280-3) contains supplementary material, which is available to authorized users.

## Introduction

Currently, metastasis is still the most challenging problem in cancer because there is no effective strategy [1]. Metastatic epidural spinal cord compression (MESCC) from spinal metastasis is a common neurological complication of advanced cancer [2]. In adults, the most common primary tumours associated with MESCC are derived from the lung, breast and prostate [2–4]. More than 90% of MESCC from lung cancers first reach the spine by direct haematogenous metastasis, usually in the vertebral column through the Baston plexus or arterial embolization or less frequently by direct invasion or extension through the intervertebral foramina, to enter the epidural space [3, 5]. Compression of the spinal cord can increase gradually as the metastasis grows into the epidural space, and acute compression may be caused by the collapse of the vertebrae [2, 3, 6]. The epidural space contains abundant epidural fat, which is found between the dura mater and vertebral wall [7, 8]. As epidural fat is the last barrier in MESCC, its role in the spinal metastasis of tumours is still unclear.

Before primary cancer cells can migrate, it is crucial for them to curate appropriate microenvironments in target organs to favour metastasis, named pre-metastatic niches [9]. One of the characteristics that define the pre-metastatic niche is organotropism, which means that certain types of cancer are predisposed to metastasize to specific organs [10, 11]. Wang et al. reported an interaction between the osteogenic pre-metastatic niche and breast cancer cells mediated by heterotypic adherens junctions to promote early-stage bone colonization [12]. Moreover, in malignant melanoma, integrin has been thought to be responsible for organ-specific metastasis, and integrin inhibitors have been considered to treat metastatic disease [13]. We currently know little about the metastatic organotropism mechanisms in metastatic tumours of the spine. Therefore, the study of this topic is crucial for MESCC treatment.

Adipose-derived mesenchymal stem cells (ADSCs), which exist widely in adipose tissue, have been shown to play an active role in tumour progression, especially metastasis [14, 15]. Many other studies have shown the complex and dynamic interaction between cancer cells and resident ADSCs [16, 17]. Our previous study revealed that ADSCs from adipose tissue adjacent to the knee joint can promote osteosarcoma proliferation and metastasis by activating the STAT3 pathway [18]. However, no research has been reported on ADSCs from epidural adipose tissue thus far, and there are many aspects that drive the need for further study of the role of epidural ADSCs in the formation and progression of MESCC.

In this study, we first isolated and cultured ADSCs from epidural adipose tissue to evaluate the role of epidural ADSCs in the interaction between primary lung cancer and MESCC. We speculated that epidural ADSCs could be activated by primary lung cancer cells to undergo myofibroblast-like differentiation, which helps primary lung cancer to form pre-metastatic niches in the spine. We examined the potential roles and related molecular mechanisms of IL-6 and STAT3 regarding the communication between tumour cells and ADSCs.

## Materials and methods

### Cell culture

Human epidural ADSCs were obtained from healthy adult donors who underwent posterior lumbar discectomy to treat lumbar fracture or lumbar disc herniation (Additional file 1: Table S1). All procedures were performed according to the ethical guidelines of the Affiliated Hospital at Qingdao University in Qingdao, China. Briefly, fresh epidural adipose tissues were collected and washed with phosphate-buffered saline (PBS) three times. Clean tissues were minced into small pieces and incubated with 0.1% collagenase (type I; Sigma-Aldrich, St. Louis, MO) in DMEM/F12 (HyClone, Thermo Scientific, USA) for 2 h at 37 °C. After gentle agitation, the tissues were added to DMEM/F12 containing 10% foetal bovine serum (FBS, Gibco, Australia) to inactivate the collagenase. After the suspension was filtered through a 100-μm mesh filter, the filtrate was centrifuged, and the cells were seeded on culture plates in culture medium (DMEM/F12, 10% FBS).

The human lung cancer cell lines PC14, A549, H3255 and H647 were purchased from the China Centre for Type Culture Collection and cultured in DMEM supplemented with 10% FBS. All cells were grown in a humidified 5% CO_2_ chamber at 37 °C.

### Characterization of ADSCs

ADSCs (passages 4–6) were analysed by flow cytometry for cellular membrane expression using CD105-PECy7, CD73-APC, CD90-FITC, CD34-PE, CD14-APC-Cy7 and CD45-PerCP-Cy5 antibodies (all from eBioscience, San Diego, CA).

The capacity of ADSCs to differentiate into osteoblasts and adipocytes was assessed as previously described [19, 20]. ADSCs were treated with an Adipogenesis Differentiation Kit and an Osteogenesis Differentiation Kit (both from Gibco, Invitrogen Corporation, Carlsbad, CA). The medium was completely changed twice per week. After 3 weeks of differentiation, the ADSCs were stained with Oil Red O and Alizarin Red S. Briefly, ADSCs were washed 3 times with PBS and then fixed in 4% paraformaldehyde (Sigma-Aldrich, St. Louis, MO) for 20 min at room temperature. The cells were then stained with 0.5% Oil Red O solution for 60 min at room temperature followed by 0.5% Alizarin Red S (both from Sigma-Aldrich, St. Louis, MO) for 20 min at room temperature. The results were assessed from images captured on an Olympus FV500 optical microscope (Olympus, Tokyo, Japan).

### Conditioned medium preparation

When the ADSCs or the four lung cancer cell lines reached 70% confluence, the medium was discarded, and the cells were further cultured in serum-free DMEM/F12 for another 24 h. The aADSCs were made by treatment with A549 CM for 12 h, and then the medium was discarded. The aADSCs were further cultured in serum-free DMEM/F12 for another 24 h. The conditioned medium of ADSCs, aADSCs and lung cancer cells was then prepared by centrifugation at 1000×*g* for 10 min and filtration through 0.22-μm filters (Millipore, Billerica, MA) for use in subsequent experiments.

### Antibody treatments

Cells were treated with 0.1 μg/mL human IL-6-neutralizing antibodies (MAB206, R&D Systems), 5 μg/mL IL-11 (MAB218, R&D Systems), 4 μg/mL leukaemia inhibitory factor (LIF)-neutralizing antibody (MAB250, R&D Systems) or an IgG control (1-001-A, R&D).

### Immunohistochemistry

ADSCs with and without lung cancer cell treatment were collected, centrifuged and fixed in 4% paraformaldehyde for 60 min. Adherent cells and tumour tissues were embedded in paraffin and cut into 4-μm sections. After the tissues were dehydrated in a graded alcohol series, antigen retrieval was performed at 4 °C using 100 μL of a solution containing rabbit monoclonal antibody against human α-SMA/FAP (1:100 dilution; ProteinTech, Chicago, IL). The diluted biotinylated secondary antibody was incubated with the sections for 20 min at 37 °C. Fresh 3,3-diaminobenzidine (DAB) solution was used to visualize the target proteins, and haematoxylin was used as a tissue counterstain. Two observers independently evaluated the expression of target proteins with an Olympus FV500 optical microscope (Olympus, Tokyo, Japan). Image-Pro Plus 5.1 was used to analyse the area and intensity of staining in five random regions (× 200 magnification) to evaluate the protein expression level.

### CCK-8 cell proliferation assay

Cell proliferation was measured using CCK-8 reagent (Dojindo, Japan). ADSCs or lung cancer cells (5000 cells/well, 5 wells/group) were seeded and cultured in 96-well plates. Cell proliferation was documented daily in accordance with the manufacturer’s protocol. CCK-8 reagent was added to each well 1.5 h before the end of the incubation period. The absorbance (OD value) at a wavelength of 450 nm was measured with a microplate reader. A colorimetric assay was performed, and growth curves were calculated using the mean results from three independent experiments.

### Cancer cell invasion assay

Each of the four lung cancer cell lines was plated in 24-well Transwell plates (Corning, NY, USA) (5 × 10^5^ cells per well). The membranes (8-μm pore diameter) in the 24-well Transwell plates were coated with 50 μL of BD Matrigel™ matrix (1:10 dilution). All cells were cultured without FBS for 24 h before the experiments. The lower chamber was filled with 600 μL of one of 2 types of culture medium: medium containing 10% FBS (control) or medium containing 10% FBS and ADSC-conditioned medium (CM). Next, the cancer cells were incubated at 37 °C for 6 h, and the cells on the lower surface of the membrane were fixed in 4% paraformaldehyde. The number of penetrating cells per high-power field was counted to represent the invasive capability of the ovarian cancer cells. All assays were performed in triplicate.

### Enzyme-linked immunosorbent assay

ADSCs (5 × 10^4^ cells per well) were cultured in 6-well plates overnight with DMEM/F12 containing 10% FBS. The supernatants of these cells were then replaced with fresh serum-free culture medium and co-cultured indirectly with one of the four lung cancer cell lines in Transwell plates with 0.4-μm pore membranes for another 24 h. The levels of IL-6, IL-11 and LIF in the supernatants were then measured using corresponding enzyme-linked immunosorbent assay (ELISA) kits (R&D Systems). The assays were performed according to the manufacturer’s instructions.

### RNA isolation and qRT-PCR assay

After treatment with lung cancer cell-CM for 48 h, ADSCs were collected, and total RNA was extracted using TRIzol reagent (Invitrogen, Carlsbad, USA). Subsequently, 2 μg of RNA was used for the synthesis of first-strand cDNA using a reverse transcription kit (Toyobo, Osaka, Japan). Quantitative real-time PCR was performed using gene-specific TaqMan probes (Applied Biosystems) and master mix (Thermo Fisher Scientific) following the manufacturer’s instructions. Gene expression was normalized to that of GAPDH. The TaqMan probes used include Acta2, Hs00426835_g1; GAPDH, Hs02786624_g1; IL6, Hs00985639_m1; IL11, Mm00434162_m1; and LIF, Hs01055668_m1.

### Western blotting

Ovarian cancer cells were lysed in NP40 buffer (Beyotime, Shanghai, China) for 10 min on ice and centrifuged at 10,000*g* to remove cell debris. Equal amounts (30 μg) of protein extracts were resolved by SDS-PAGE and then transferred to polyvinylidene fluoride (PVDF) membranes (Bio-Rad, Hercules, CA). The transferred membranes were incubated with primary sheep monoclonal antibody against human FAP (1:1000 dilution; R&D Systems); mouse monoclonal antibody against human E-cadherin and N-cadherin (1:1000; Cell Signaling Technology); rabbit monoclonal antibody against human α-SMA, MMP2 and MMP9 (1:1000; Cell Signaling Technology); and rabbit antibody against β-actin (1:500 dilution; ProteinTech, Chicago, IL). The membranes were then incubated with peroxidase-conjugated AffiniPure secondary IgG antibodies (H+L) (1:2000; R&D Systems). With a chemiluminescence detection system, the protein-antibody complexes were detected and quantitated using Image Lab™ version 5.1 software (Bio-Rad, Hercules, CA).

### Statistical analysis

Our data are expressed as the mean ± standard deviation, and we calculated the means from at least three independent experiments. The significance of the differences was analysed with a two-tailed Student’s *t* test or a one-way analysis of variance. A value of *P* < 0.05 was considered to indicate significance.

## Results

### Identification of epidural ADSCs and A549 cells activates phenotypic changes in ADSCs

**Fig. 1:**
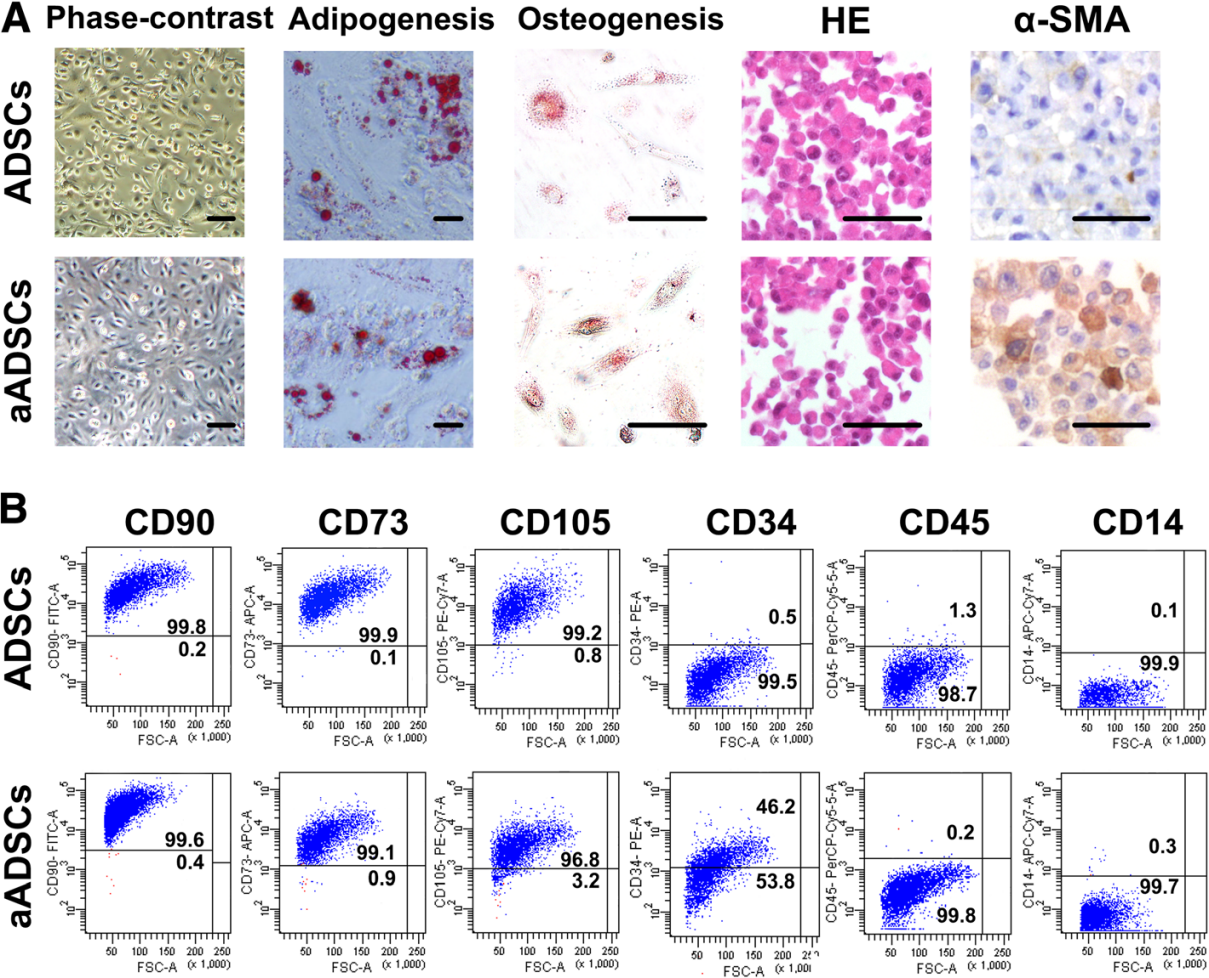
Isolation and characterization of ADSCs derived from epidural adipose tissue. **a** Representative photomicrographs of primary epidural ADSCs cultured alone or in the presence of conditioned medium (CM) from lung cancer cells after reaching confluence at passage 5. Osteogenic and adipogenic differentiation was examined in ADSCs at passage 5. Cells were fixed with 4% formalin and stained with Alizarin Red to assess the osteogenic differentiation and Oil Red O dye to visualize lipid droplets that result from adipogenic differentiation. H&E staining and immunohistochemical analysis demonstrate the expression of the myofibroblast marker α-SMA in ADSCs. Scale bar = 100 μm. **b** Flow cytometry analysis of the positive markers CD90, CD105 and CD73 and the negative markers CD34, CD45, and CD14 for determining mesenchymal marker expression in isolated ADSCs. aADSCs, ADSCs treated with lung cancer cell-conditioned medium containing α-SMA and FAP

Primary epidural ADSCs cultured with or without CM from the lung cancer cell line A549 displayed fibroblastic-like adherence and typical spindle-shaped morphology (Fig. 1a). The stemness of the two types of epidural ADSCs was verified by examining their ability to undergo adipogenic and osteogenic differentiation in vitro (Fig. 1a). In addition, both ADSCs cultured alone and those treated with A549 cell CM positively expressed characteristic mesenchymal surface markers (CD90, CD73 and CD105) and showed lower expression of haematopoietic cell markers (CD34, CD45 and CD14) (Fig. 1b). However, ADSCs treated with A549 cell CM expressed a much lower level of the mesenchymal marker CD105 (Fig. 1b). Immunohistochemistry assays indicated that epidural ADSCs treated with A549 cell CM expressed higher levels of α-SMA than did ADSCs cultured in an untreated medium (Fig. 1a).

### Lung cancer cells induce phenotypic changes in epidural ADSCs

**Fig. 2:**
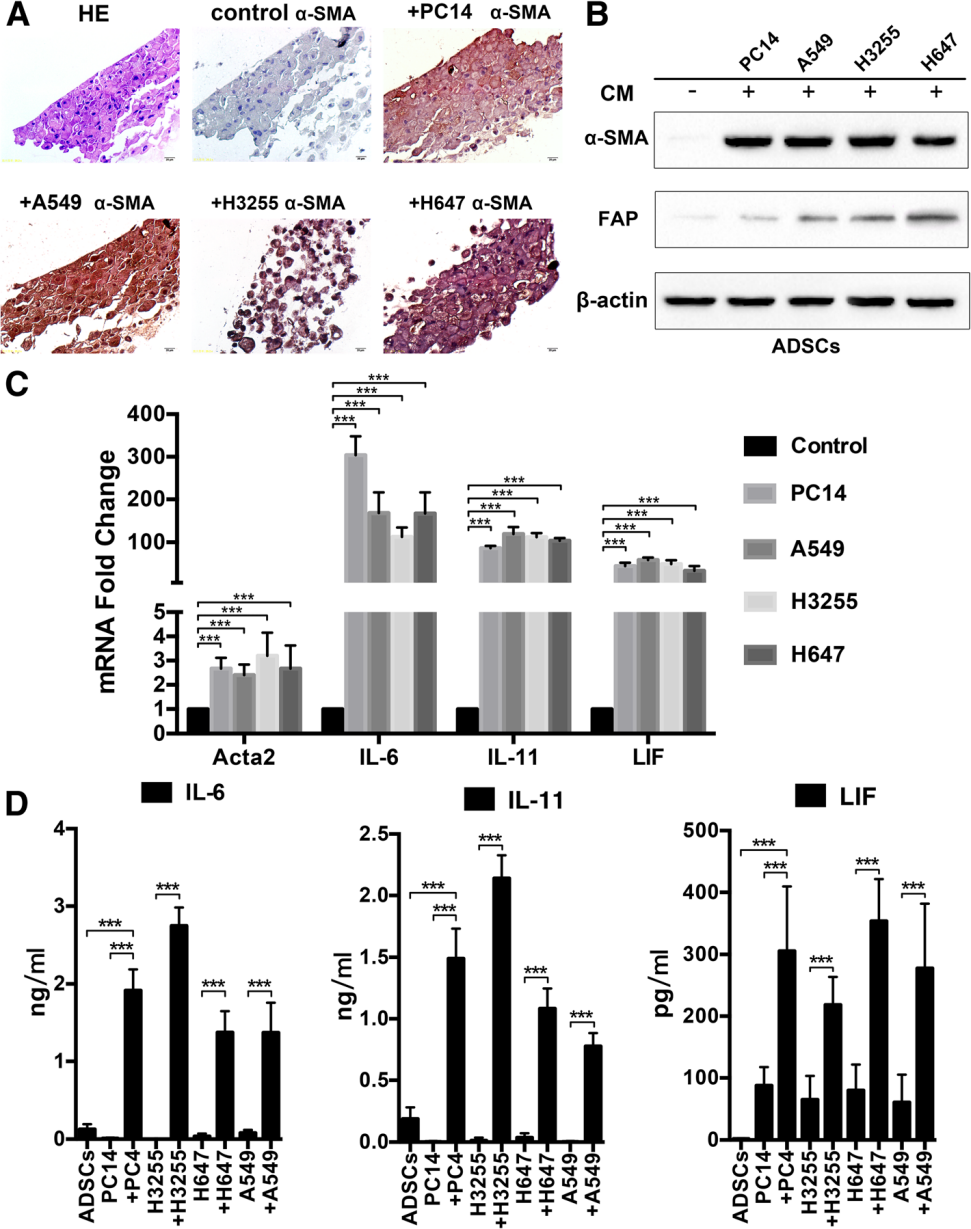
Lung cancer cells induce ADSCs to express myofibroblast markers accompanied by the secretion of IL-6 family cytokines. **a** H&E staining and immunohistochemical analysis demonstrate the expression of the myofibroblast marker α-SMA in epidural ADSCs. ADSCs treated with CM from one of four lung cancer cell lines for 48 h expressed high levels of α-SMA. Scale bar = 100 μm. **b** Western blotting analysis of α-SMA and FAP expression in epidural ADSCs treated with CM from one of four lung cancer cell lines for 48 h. ADSCs cultured in untreated medium served as controls. **c** qRT-PCR analysis of IL-6, IL-11, LIF and Acta2 mRNA expression in epidural ADSCs treated with CM from one of four lung cancer cell lines for 48 h. ADSCs cultured in untreated medium served as controls. **d** ELISA of IL-6, IL-11 and LIF in supernatants of ADSCs treated with lung cancer cell CM for 24 h. The supernatants of the four lung cancer cell lines and ADSCs cultured in untreated medium served as controls. ***P < 0.001

To further characterize the role of lung cancer cells in the phenotypic changes in epidural ADSCs, we treated epidural ADSCs with CM from four lung cancer cell lines, PC14, A549, H3325 and H647, and evaluated the expression of α-SMA, a marker of myofibroblasts [21, 22]. The expression of α-SMA in CM-treated ADSCs was examined by immunohistochemistry (Fig. 2a), and epidural ADSCs cultured in an untreated medium were used as controls. Compared with their controls, nearly all CM-treated ADSCs expressed elevated levels of α-SMA (*P* < 0.001). Moreover, western blot analysis of FAP, another myofibroblast marker, and α-SMA expression in epidural ADSCs treated with four types of lung cancer cell CM for 2 days revealed that CM treatment markedly elevated α-SMA and FAP expression in epidural ADSCs (Fig. 2b).

### Epidural ADSCs secreted IL-6 family cytokines during myofibroblast differentiation

Following exposure of ADSCs to lung cancer cell CM for 2 days, myofibroblast marker genes were upregulated, suggesting that cancer cells mediate myofibroblast differentiation of normal epidural ADSCs. Since myofibroblasts secrete many factors, such as the IL-6 family of cytokines (which have been shown to play a role in tumour progression [23–25]), we performed qRT-PCR to analyse the expression of IL-6, IL-11 and LIF, the three main members of the IL-6 family, and of the α-SMA gene Acta2 in myofibroblasts differentiated from epidural ADSCs. Treatment with CM from 4 different lung cancer cell lines for 48 h led to increased expression of Acta2, IL-6, IL-11 and LIF (Fig. 2c). An ELISA was performed to confirm and quantify the elevated secretion of IL-6, IL-11 and LIF during the differentiation of epidural ADSCs (Fig. 2d). These results suggested that treatment of lung cancer cell CM stimulated the production and secretion of cytokines from epidural ADSCs.

### Autocrine IL-6 activates STAT3 signalling in differentiated epidural ADSCs

**Fig. 3:**
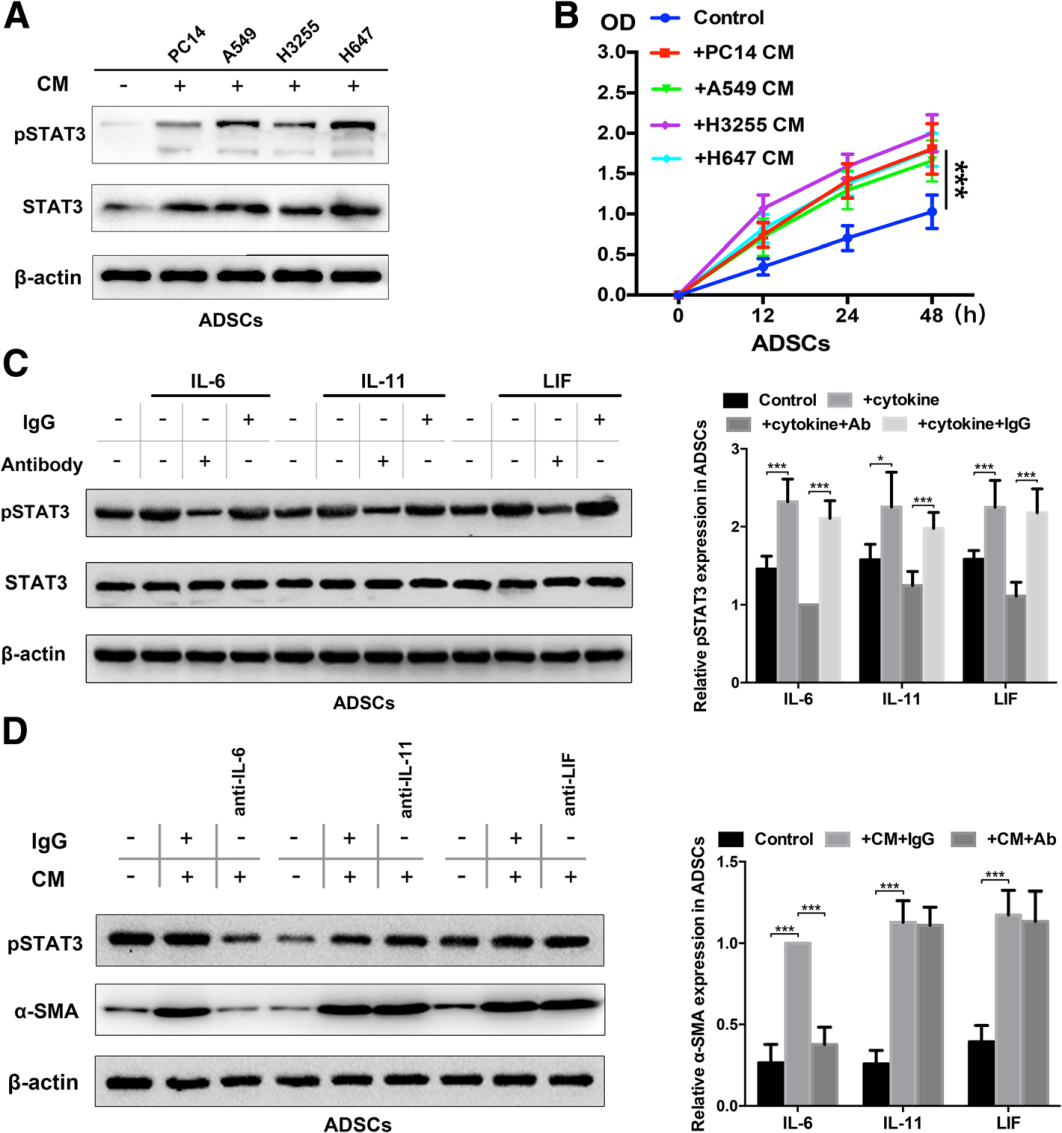
Autocrine IL-6 activates STAT3 signalling in lung cancer cell-induced epidural ADSCs. **a** Epidural ADSCs were pre-treated with CM from lung cancer cells for 48 h, and pSTAT3 and STAT3 expression levels were detected by western blotting. Epidural ADSCs cultured in untreated medium served as a control. **b** The effects of lung cancer cell CM on epidural ADSC proliferation were evaluated using the CCK-8 assay. Epidural ADSCs were treated with CM from one of four lung cancer cell lines, and the optical density of both groups at 450 nm was analysed. Data from three separate experiments are shown. **c** Western blot analysis of pSTAT3 and STAT3 in epidural ADSCs treated with either 10 ng/mL recombinant IL-6, 10 ng/mL recombinant IL-11 or 50 ng/mL recombinant LIF in the presence or absence of either neutralizing antibodies or isotype controls. Loading control, actin. **d** Western blot analysis of pSTAT3 and α-SMA expression in epidural ADSCs treated with lung cancer cell CM in the presence or absence of neutralizing antibodies against IL-6, IL-11 or LIF. Loading control, actin. *P < 0.05; **P < 0.01; ***P < 0.001

The IL-6 family cytokines are known activators of signal transducer and activator of transcription (STAT) factors, most notably the STAT3 signalling pathway, which plays key roles in cell growth and proliferation [26]. We thus examined whether the STAT3 signalling pathways activated by IL-6 family cytokines contributed to phenotypic changes in epidural ADSCs. We first assessed the activation of the STAT3 pathway in response to CM from four lines of lung cancer cells and observed that STAT3 signalling was activated in quiescent ADSCs from healthy donors exposed to lung cancer cell CM for 2 days (Fig. 3a). Moreover, CM from lung cancer cells significantly promoted the proliferation of ADSCs (Fig. 3b). To confirm that STAT3 signalling was activated in epidural ADSCs in response to paracrine secretion from cancer cells, we evaluated STAT3 phosphorylation in ADSCs after the addition of recombinant IL-6, IL-11 and LIF by western blot. The expression of STAT3 phosphorylation in ADSCs was significantly elevated by all three cytokines, and these effects were prevented by the treatment with neutralizing antibodies against each cytokine (Fig. 3c). Interestingly, the IL-6-neutralizing antibody exerted the most prominent effect.

To determine which cytokines activate the STAT3 pathway in epidural ADSCs in an autocrine manner, we exposed ADSCs to A549 cell CM in which IL-11, IL-6 or LIF had been neutralized by antibodies and assessed which neutralizing antibody blocked STAT3 pathway activation (Fig. 3d). Our data confirmed that IL-6 has a major role in the activation of STAT3 signalling in ADSCs. In contrast, neutralization of either IL-11 or LIF did not reduce the expression levels of pSTAT3. Altogether, these results suggest that epidural ADSC-derived IL-6 secretion induces STAT3 signalling activation downstream and promotes the myofibroblast phenotype of ADSCs in an autocrine manner.

### ADSCs activated by lung cancer cells trigger the proliferation and invasion of lung cancer cells in vitro

**Fig. 4:**
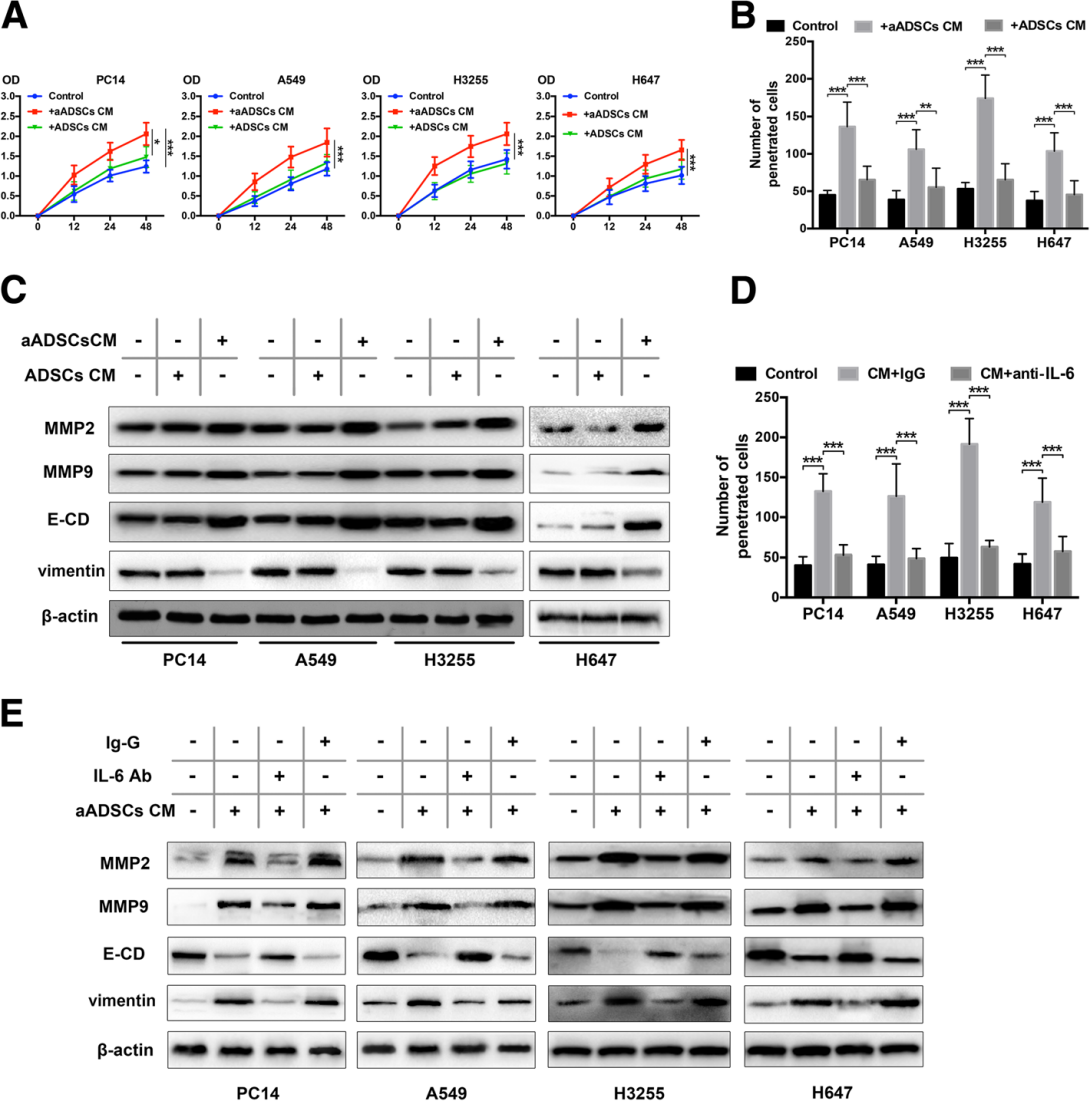
Activated ADSCs trigger the proliferation and invasion of lung cancer cells by regulating MMP2/9 expression and EMT. **a** The effects of activated ADSCs on lung cancer cell proliferation were evaluated using the CCK-8 assay. Four lung cancer cell lines were cultured in the presence of ADSC-CM or aADSC-CM, and the optical density at 450 nm was analysed. The cancer cells cultured alone in normal growth medium were used as negative controls. Data from three separate experiments are shown. **b** The number of lung cancer cells that migrated through 8-μm Transwell membrane pores was counted to determine the changes in the invasive capabilities in response to CM from epidural ADSCs or aADSCs. **c** MMP2/9, E-cadherin and vimentin expression levels in four lung cancer cell lines treated with ADSC-CM or aADSC-CM were examined by western blotting. Lung cancer cells cultured in an untreated medium served as controls. **d** Lung cancer cells were treated with neutralizing antibodies against IL-6, isotype controls and aADSC-CM. Lung cancer cell invasion was analysed using a Transwell assay. Lung cancer cells cultured with untreated medium served as negative controls. **e** Lung cancer cells were treated with neutralizing antibodies against IL-6, isotype controls and aADSC-CM. MMP2/9, E-cadherin and vimentin expression levels in four lung cancer cell lines were analysed using western blotting. Lung cancer cells cultured with untreated medium served as negative controls. ***P < 0.001

As myofibroblasts are well-known stromal cells in the tumour microenvironment and regulate tumour cell invasion and proliferation [27], we investigated the proliferative kinetic effect of activated epidural ADSCs on four lung cancer cell lines. We have shown that all four lines of lung cancer cells can activate ADSCs, and we chose to use A549 with distinct activated effects to activate ADSCs (aADSCs). We examined the proliferation of lung cancer cells cultured with CM from ADSCs and aADSCs for 2 days. Lung cancer cells cultured with untreated medium were used as controls. The cell proliferation activity increased significantly after treatment with aADSC-CM for 48 h (all *P* < 0.001, Fig. 4a) compared with that in the control and ADSC groups.

To further determine whether the myofibroblast-like differentiated ADSCs were associated with the increased aggressiveness of lung cancer cells, we performed Transwell invasion assays with the four lung cancer cell lines treated with ADSC-CM or aADSC-CM for 48 h. Cancer cells cultured in an untreated medium were used as a control. The presence of aADSC-CM elevated the number of invading EOC cells compared with that in the control group, and a two- to fourfold increase was observed (all *P* < 0.001) (Fig. 4b). There was no significant effect of ADSC-CM on the invasion of cancer cells. These data suggest that, like myofibroblasts, lung cancer cell-mediated myofibroblast-like differentiation in epidural ADSCs also promotes tumour progression.

### Lung cancer EMT and MMP expression induced by differentiated ADSCs

Epithelial-mesenchymal transition (EMT), which is characterized by the loss of the epithelial marker E-cadherin and the gain of the mesenchymal marker vimentin, plays important roles in tumour progression, including intravasation, metastasis and resistance to therapy [28–30]. Matrix metalloproteases (MMPs) degrade ECM proteins and influence cell proliferation and have been associated with tissue remodelling during various physiological processes [31, 32]. In cancer, elevated MMP levels play a role in tumour progression and invasiveness. As an important cytokine, IL-6 has been observed to regulate EMT in cancers [33, 34]; therefore, we performed western blotting to detect MMP2/9, E-cadherin and vimentin expression in lung cancer cells treated with ADSC-CM or aADSC-CM. The four lung cancer cell lines treated with aADSC-CM exhibited a significant increase in MMP2/9 and vimentin expression and a decrease in E-cadherin expression (Fig. 4c). Therefore, our results suggested that EMT of lung cancer cells was induced by differentiated ADSCs and corresponded to elevated expression of MMPs.

### IL-6 mediated the cancer-promoting effect of differentiated ADSCs

Next, we examined the effects of IL-6 on lung cancer EMT and MMP expression regulation in differentiated ADSCs. The number of penetrating lung cancer cells was significantly increased after treatment with aADSC-CM and was reduced when cancer cells were cultured with IL-6-neutralizing antibody, confirming that IL-6 is essential for the invasion-promoting effect of aADSCs (Fig. 4d). To corroborate a role for IL-6 in regulating lung cancer cell proliferation, we performed the CCK-8 assay on cancer cells exposed to aADSC-CM in the presence or absence of IL-6 antibody. Indeed, the enhancing effects of aADSCs on the proliferation of cancer cells were blocked by the IL-6 antibody (Fig. 4d). Furthermore, IL-6 inhibition dramatically suppressed MMP2/9 and vimentin expression but elevated E-cadherin expression all four lung cancer cell lines treated with aADSCs-CM (Fig. 4e).

## Discussion

Spinal metastasis remains a major challenge in cancer, and in patients with lung cancer, MESCC could be observed without a pre-existing cancer diagnosis [3, 4, 35]. Before primary cancer cells can metastasize, it is necessary to prepare a hospitable environment in the secondary organ for the colonization and survival of circulating tumour cells. Epidural fat has been described as a semifluid tissue and has been associated with low back pain, canal stenosis and the distribution of drugs injected into the epidural space [36, 37]. A pre-metastatic niche such as epidural fat is a fertile microenvironment and forms an inflammatory and high-energy environment after carcinogenesis, which may partly explain the metastatic organotropism in patients with lung cancer.

In our study, we isolated ADSCs from epidural adipose tissues to investigate the ability of lung cancer cells to induce a phenotypic transformation in normal epidural ADSCs. We found that ADSCs treated with lung cancer cell CM expressed the myofibroblast markers α-SMA and FAP. Furthermore, another approach to identify lung cancer-treated ADSCs is detecting the secretion of IL-6 family cytokines, including IL-6, IL-11 and LIF, as initiators of the cytokine cascade that leads to STAT3 activation. Notably, we identified that IL-6 activates the STAT3 pathway in epidural ADSCs in an autocrine manner. Additionally, similar to our previous work [18], the tumour-promoting effects of activated epidural ADSCs were observed in lung cancer cells via the regulation of MMP expression and EMT. Accordingly, IL-6 inhibition in ADSCs significantly antagonized the tumour-promoting effects of these cells. We demonstrated that normal epidural ADSCs could undergo myofibroblast-like differentiation and support the metastasis of lung cancer, which may be a contributing factor to the formation of a pre-metastatic niche.

Previous studies have reported many cellular components that contribute to the formation of pre-metastatic niche, including fibroblasts, osteoclasts, bone marrow-derived cells (BMDCs), endothelial cells and hepatic stellate cells [9]. The recruitment of osteoclast-specific microRNAs and osteoclastogenesis can be regulated by tumour cells, which is important for bone tumour metastasis [38]. In addition, fibroblasts in the primary tumour stroma could drive the selection of organ-specific metastatic cancer cells that are primed for metastasis to bone [39]. In particular, fibroblasts have been shown to induce the expression of inflammatory factors and growth factors, such as IL-6, TGF-β and MMPs, in the pre-metastatic niche [40, 41]. Furthermore, within the pre-metastatic bone marrow, recruited BMDCs remodel the microenvironment to support tumour cell survival and proliferation and promote tumour metastasis through secretory factors [42–44].

Various studies have shown that aberrant expression of the IL-6 family of cytokines, especially IL-6 and IL-11, is correlated with high tumour burden and is considered one of the most important cytokine families during tumourigenesis and metastasis [24, 45, 46]. This family exerts its activity by stimulating the cellular components in the tumour microenvironment and in cancer stem-like cells, which regulate pathways that promote EMT of tumour cells, propelling them to migrate via the STAT3 pathway [47, 48]. The IL-6 secretory phenotype of ADSCs co-cultured with cancer cells has been observed in many studies and is accompanied by elevated cancer cell proliferation and metastasis, which suggests a role for ADSCs in promoting tumour proliferation and invasive properties [15, 49, 50]. There are at least two benefits to targeting IL-6: depleting activated ADSCs in the epidural space would reduce the secretion of tumour-promoting cytokines and chemokines to ameliorate tumour metastasis, and shifting activated ADSCs to a more undifferentiated state would restore the barrier effect against metastasis. Based on this premise, blocking IL-6 signalling may provide a potential approach to developing novel therapies.

## Conclusions

Our results support the notion that ADSCs in epidural adipose tissues can be activated by lung cancer cells and are involved in the formation of pre-metastatic niches in the spine, which promotes cancer growth and metastasis by elevating MMP expression and inducing EMT of lung cancer cells. Our study thus explains the role of IL-6 autocrine signalling in activating the STAT3 pathway in ADSCs and the effects of blocking IL-6 on the tumour-promoting activity of activated epidural ADSCs. However, our knowledge regarding the exact mechanism by which epidural ADSCs in pre-metastatic niches influence lung cancer metastasis and progression remains limited. This work could direct the design of treatment strategies meant to convert potential tumour-promoting epidural ADSCs into a passive state in the spinal canal.

## Additional file

Additional file 1: **Table S1.** Epidural adipose tissue donor information. (DOCX 496 kb)

## Data Availability

The datasets used and/or analysed during the current study are available from the corresponding author on reasonable request.

## Abbreviations

ADSCs: Adipose-derived mesenchymal stem cells
CM: Conditioned medium
EMT: Epithelial-mesenchymal transition
FAP: Fibroblast activation protein
FBS: Foetal bovine serum
IL-6: Interleukin-6
MESCC: Metastatic epidural spinal cord compression
MMP: Matrix metalloproteinases
PBS: Phosphate-buffered saline
STAT3: Signal transducer and activator of transcription 3
α-SMA: α-Smooth muscle actin
