# Supplementary material for: Epidural adipose tissue-derived mesenchymal stem cell activation induced by lung cancer cells promotes malignancy and EMT of lung cancer
Source: Stem Cell Res Ther. 2019 Jun 13;10:168. doi: 10.1186/s13287-019-1280-3 (PMC6567486; doi:10.1186/s13287-019-1280-3)
Supplement: Supplementary file 1 — Table S1. Epidural adipose tissue donor information. (DOCX 496 kb) [file 13287_2019_1280_MOESM1_ESM.docx]

Table S1 Epidural Adipose Tissue Donor Information

| Dornor  (n) | Age  (Years) | Height  (cm) | Weight  (Kg) | BMI  (Kg/m^2^) | Gender (M/F) | Comments | Operation |
| --- | --- | --- | --- | --- | --- | --- | --- |
| 1 | 49 | 172 | 73 | 24.6 | M | Lumbar fracture | Posterior lumbar decompression and pedicle screw fixation |
| 2 | 54 | 175 | 80 | 26.1 | F | Lumbar disc herniation | Posterior lumbar discectomy |
| 3 | 40 | 176 | 82 | 26.4 | F | Lumbar disc herniation | Posterior lumbar discectomy |

Note: Epidural adipose tissue was harvested from patients with lumbar fracture and lumbar disc herniation, undergoing posterior lumbar decompression or lumbar discectomy. BMI and comments are essential.
